# Predictive structured–unstructured interactions in EHR models: A case study of suicide prediction

**DOI:** 10.1038/s41746-022-00558-0

**Published:** 2022-01-27

**Authors:** Ilkin Bayramli, Victor Castro, Yuval Barak-Corren, Emily M. Madsen, Matthew K. Nock, Jordan W. Smoller, Ben Y. Reis

**Affiliations:** 1 Predictive Medicine Group, Computational Health Informatics Program, Boston Children’s Hospital, Boston, MA USA; 2 Harvard University, Cambridge, MA USA; 3 Mass General Brigham Research Information Science and Computing, Boston, MA USA; 4 Department of Psychiatry, Massachusetts General Hospital, Boston, MA USA; 5 Psychiatric and Neurodevelopmental Genetics Unit, Center for Genomic Medicine, Massachusetts General Hospital, Boston, MA USA; 6 Center for Precision Psychiatry, Department of Psychiatry, Massachusetts General Hospital, Boston, MA USA; 7 Department of Psychology, Harvard University, Cambridge, MA USA; 8 Mental Health Research Program, Franciscan Children’s, Brighton, MA USA; 9 Harvard Medical School, Boston, MA USA

**Keywords:** Translational research, Epidemiology

## Abstract

Clinical risk prediction models powered by electronic health records (EHRs) are becoming increasingly widespread in clinical practice. With suicide-related mortality rates rising in recent years, it is becoming increasingly urgent to understand, predict, and prevent suicidal behavior. Here, we compare the predictive value of structured and unstructured EHR data for predicting suicide risk. We find that Naive Bayes Classifier (NBC) and Random Forest (RF) models trained on structured EHR data perform better than those based on unstructured EHR data. An NBC model trained on both structured and unstructured data yields similar performance (AUC = 0.743) to an NBC model trained on structured data alone (0.742, *p* = 0.668), while an RF model trained on both data types yields significantly better results (AUC = 0.903) than an RF model trained on structured data alone (0.887, *p* < 0.001), likely due to the RF model’s ability to capture interactions between the two data types. To investigate these interactions, we propose and implement a general framework for identifying specific structured-unstructured feature pairs whose interactions differ between case and non-case cohorts, and thus have the potential to improve predictive performance and increase understanding of clinical risk. We find that such feature pairs tend to capture heterogeneous pairs of general concepts, rather than homogeneous pairs of specific concepts. These findings and this framework can be used to improve current and future EHR-based clinical modeling efforts.

## Introduction

**Fig. 1:**
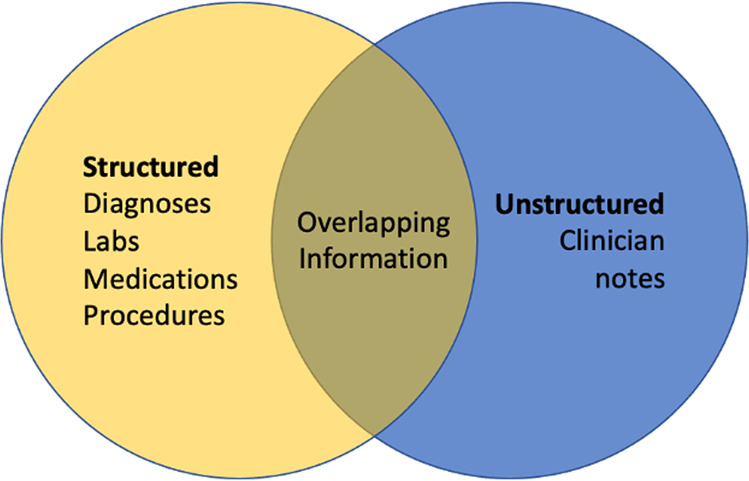
Information overlap in EHR data. Electronic health records contain both structured and unstructured data. These two types of data contain both unique and overlapping information.

In recent years there has been a proliferation of clinical prediction models powered by electronic health records (EHRs). Many prediction models rely primarily on structured data from the EHR, which typically includes diagnostic, laboratory, medication, and procedure codes. Yet most EHRs also contain unstructured data such as clinician notes, which may include information already captured in the structured data, as well as information not present in the structured data (Fig. 1). Unstructured EHR data have been used for clinical predictive tasks, both as a standalone feature-set and in combination with structured data^1–4^.

In order to optimally integrate both structured and unstructured data and improve predictive performance, it is important to understand the predictive value of each data type. It is also important to understand the interactions between these two data types and identify instances where the nature of these interactions differs between case and non-case populations. Such differences can be valuable for deepening our understanding of clinical risk and for improving clinical risk prediction in models that are able to capture these interactions.

As a case study, we focus on suicide prediction. Approximately 800,000 people die by suicide every year worldwide, accounting for 1.5% of all deaths^5^. Suicide is the tenth leading cause of death in North America and a leading cause of death globally among persons 15–24 years of age^6^. With suicide-related mortality rates rising in recent years^7^, it is becoming increasingly urgent to understand, predict, and prevent suicidal behavior. Early and accurate identification of individuals with elevated risk for suicide attempts is critical for developing effective suicide prevention strategies. Predicting suicide risk, however, is a complex challenge. The intuition of clinicians for detecting at-risk individuals is no better than random chance^8^, underscoring the potential value of algorithmic approaches to this challenge.

In recent years, rapidly growing quantities of electronic health data along with advancements in statistical learning methods have enabled the development of suicide risk prediction models. We recently developed one such model using data from over 1.7 million patients in a large healthcare system (Mass General Brigham)^9^; the model detected 45% of suicide attempts an average of 3–4 years in advance, with a specificity of 90% and an area under the receiver operating curve (AUC) of 0.77. Since structured EHR data capture only some elements of clinical presentation, in the present study, we seek to improve upon this prediction accuracy by examining features extracted using natural language processing (NLP) from unstructured clinician notes. (For simplicity, we refer to these as “unstructured features”.)

The goals of this study are threefold: (1) To compare the predictive value of structured and unstructured EHR data as standalone datasets for predicting suicide risk; (2) to evaluate the increase in prediction performance when integrating both structured and unstructured data using various models; and (3) to identify structured-unstructured feature pairs in which the interaction between the two features differs substantially between case and non-case populations, and which may thus have the potential to improve predictive performance. To achieve the latter, we propose a framework for identifying structured-unstructured feature pairs in which the interaction between the two features differs significantly between case and non-case cohorts.

## Results

### Study population

**Fig. 2:**
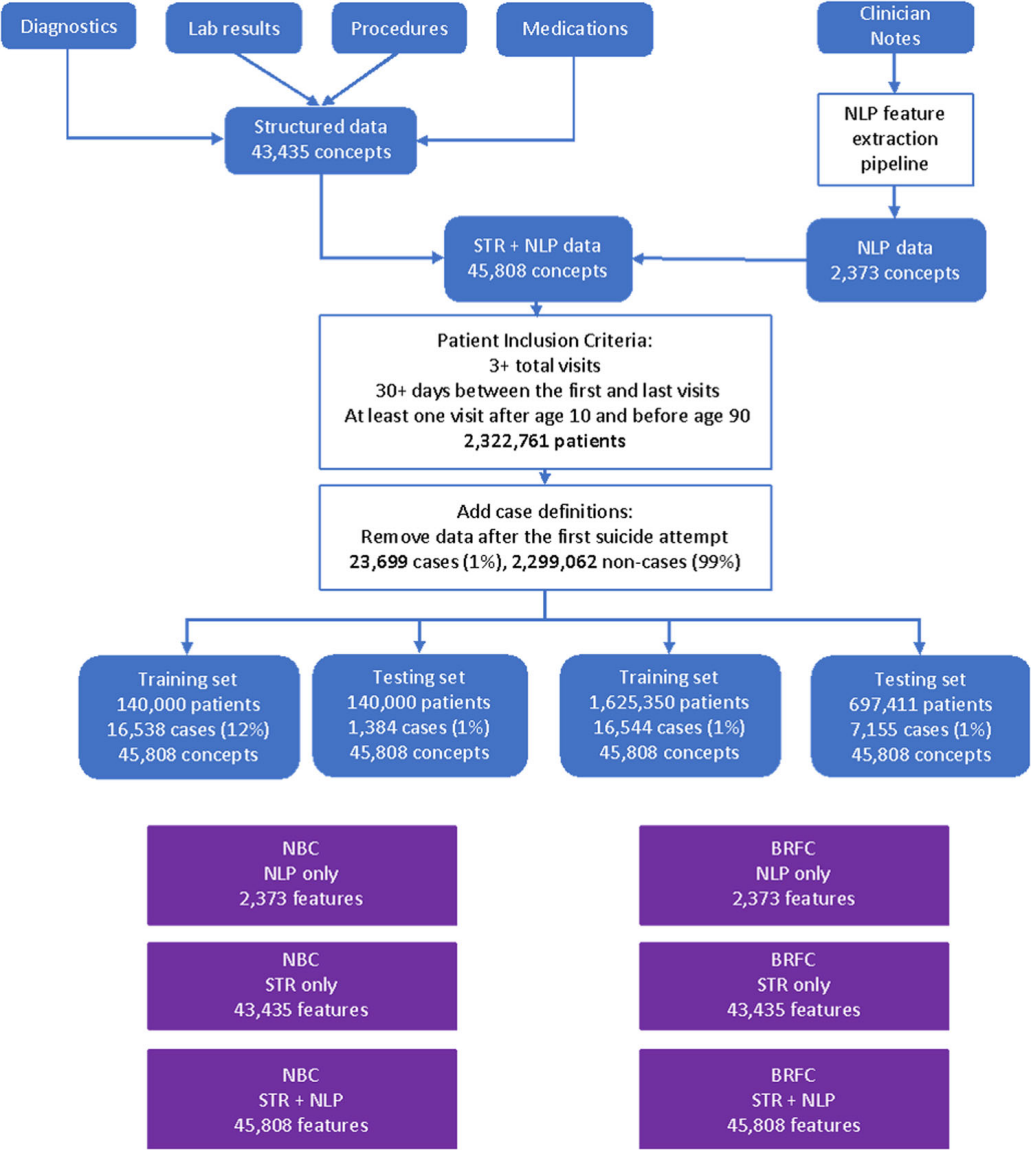
Data and modeling workflow. The diagram describes the filtering and processing steps taken to arrive at the final datasets used for training and testing different models described in this paper. STR—Structured Data; NLP—Unstructured data processed by Natural Language Processing; NBC—Naïve Bayesian Classifier; BRFC—Balanced Random Forest Classifier.

**Fig. 3:**
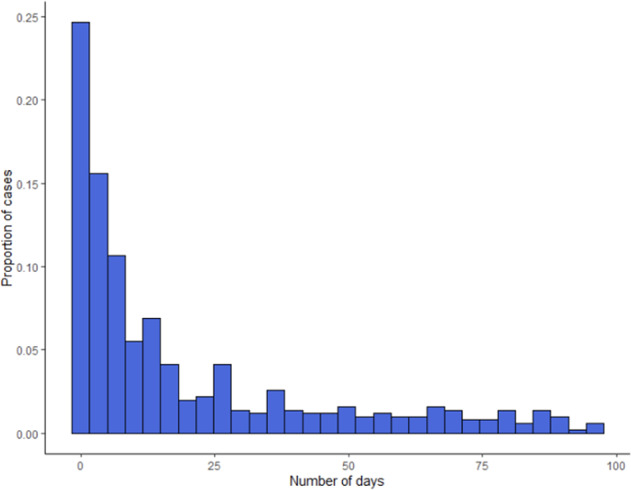
Distribution of time between penultimate hospital visit and first suicide attempt, in days. As the distribution was highly skewed, the *x*-axis was capped at 100 days for clarity. A few patients had several years between their last recorded visit and suicide attempt.

Approximately 2,303,376 individuals did not have sufficient number of visits to be included in the analysis. Of these, 11,316 had a suicide attempt (0.5%). Many of the excluded individuals had a single emergency department visit, hospitalization, or medical concept recorded over the 20 year course of the dataset. Applying the inclusion and exclusion criteria to the extracted data yielded 1,625,350 training subjects for the NBC models, which included 1,608,806 non-cases (99%) and 16,544 cases (1%) (Fig. 2). The testing set consisted of 697,411 subjects, including 7,155 cases. For the BRFC models, the dataset included 140,000 subjects for each of the training and testing populations, with the former having 16,538 cases (12%, due to the sampling approach mentioned above) and the latter having 1,384 (1%, reflecting the prevalence in the clinical population). For case subjects, the median time between the penultimate hospital visit and first suicide attempt was 35 days and the mean was 286 days. Figure 3 shows the distribution of time between the penultimate visit and first suicide attempt.

For both experiments, we had the same set of 45,808 features which included 43,435 structured features (95%) and 2,373 features derived from unstructured data using NLP (5%). Table 1 shows the correspondence between structured and unstructured codes for several sample concepts.

**Table 1:** Correspondence between structured and unstructured codes.

Concept	Struct.	Unstruct.	Both	Total
Impulse control disorder	145 (19%)	688 (86%)	37 (5%)	796
Unspecified bipolar disorder	1,322 (30%)	4,053 (94%)	1,051 (24%)	4,324
Schizo-affective disorder	250 (42%)	522 (88%)	177 (30%)	595
Opioid dependence or abuse	1,183 (27%)	3,893 (90%)	761 (17%)	4,315

The number of patients that have a structured EHR code for a given concept (first column), an NLP code (based on a free-text mention of that concept in their unstructured clinician notes, second column), and both a structured code and an NLP code for the given concept. Since NLP concepts are more general, each row includes one NLP code but several structured codes with similar descriptions. Furthermore, “opioid dependence” and “opioid abuse” codes were merged into one code since many EHR codes mention both opioid dependence and abuse.

### Model performance

**Fig. 4:**
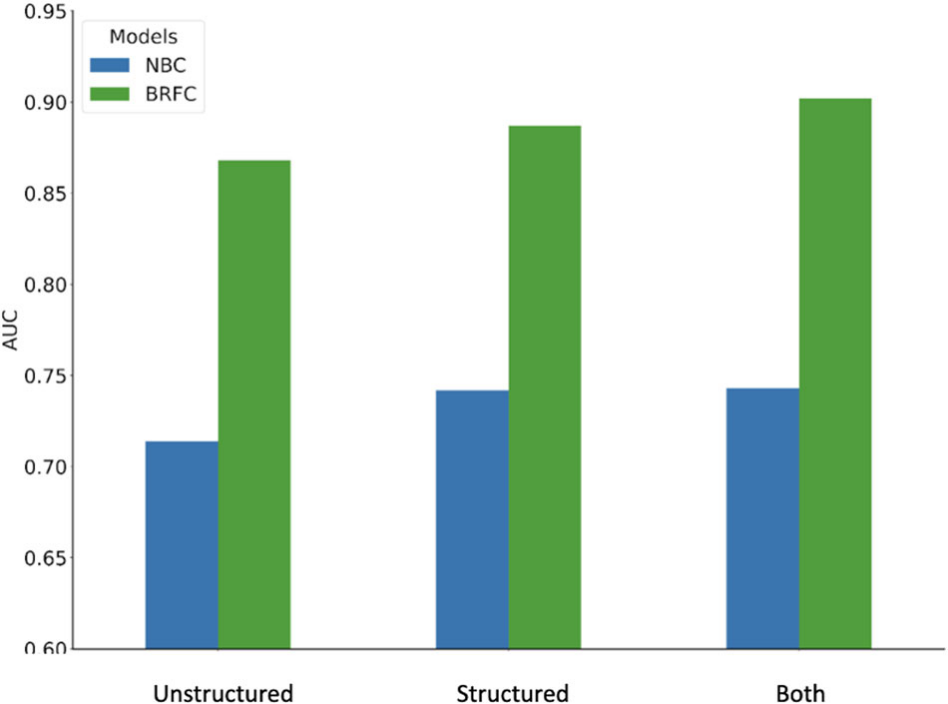
Performance of NBC and BRFC models, by type of data used. BRFC models perform considerably better than NBC models in terms of AUC across all three datasets. Combining structured and unstructured data yields better performance than using structured data alone, which itself performs better than using unstructured data only.

The results of training and testing are presented in Tables 2 and 3 and Fig. 4. We found that for both NBC and BRFC modeling approaches, training on structured data features resulted in higher predictive performance than training on features derived from unstructured data, with an improvement in AUC of 2–3% (*p* < 0.001).

**Table 2:** Performance of NBC models on the test set.

	Unstructured		Structured		Both	
Specificity	PPV	Sensitivity	PPV	Sensitivity	PPV	Sensitivity
**0.99**	0.070	0.079	0.072	0.076	0.088	0.092
**0.95**	0.046	0.254	0.047	0.239	0.051	0.260
**0.90**	0.035	0.378	0.036	0.365	0.039	0.391
**0.80**	0.024	0.520	0.026	0.530	0.027	0.540
**AUC**	0.714		0.742		0.743	

There is no significant increase (*p* = 0.688) in AUC between the model based on structured-data-only and the model based on both structured and unstructured data.

**Table 3:** Performance of BRF models on the test set.

	Unstructured		Structured		Both	
Specificity	PPV	Sensitivity	PPV	Sensitivity	PPV	Sensitivity
**0.99**	0.142	0.168	0.191	0.246	0.219	0.267
**0.95**	0.082	0.447	0.092	0.507	0.097	0.545
**0.90**	0.057	0.608	0.063	0.657	0.066	0.697
**0.80**	0.037	0.766	0.040	0.820	0.041	0.845
**AUC**	0.868		0.887		0.902	

There is a significant increase (*p* < 0.001) in AUC between the model based on structured-data-only and the model based on both structured and unstructured data. There are also substantial increases in sensitivity.

For the NBC model, training on *both* structured and unstructured data yielded no significant improvement over training on structured data alone (*p*-value = 0.67). However, for BRFCs, training with both structured and unstructured data led to a moderate but significant 1.6% increase in AUC relative to training on structured data alone (*p*-value < 0.001). The combined structured and unstructured BRFC model also exhibited moderate increases in PPV and sensitivity from the structured-data-only model across all specificity thresholds, with a 4% increase in sensitivity at both 0.90 and 0.95 specificity, in addition to increases in PPV.

### Contingency analysis

Tables 4 and 5 shows structured-unstructured feature pairs in which the relationship between the two features differed most between case and non-case cohorts—namely, those with the highest interaction heterogeneity. Table 4 shows feature pairs in which the structured feature *A* was associated with greater suicide risk (i.e., feature *A* occurred more frequently in the case cohort than in the non-case cohort). These include drug and opioid use, suicidal ideation, and borderline personality disorder which are associated with various high-risk NLP features including schizophrenia, self-reported suicide attempts, imprisonment, and homelessness.

**Table 4:** Structured-unstructured feature pairs *AB* with high interaction heterogeneity (IH), where *A* is a strong *risk factor* for suicide attempt.

Features		Cases				Non-cases				
Structured (*A*)	Unstructured (*B*)	*A*	*B*	*AB* expected	*AB* actual	*A*	*B*	AB expected	*AB* actual	IH
Other, mixed, or unsp. Drug abuse, unsp. Use	Suicide attempts	2356	3741	374.53	1003	148	563	3.54	53	77.55
Other, mixed, or unsp. Drug abuse, unsp. Use	Section XII	2356	3045	304.85	849	148	403	2.53	43	74.72
Other, mixed, or unsp. Drug abuse, unsp. Use	Living on the street	2356	1113	111.43	532	148	154	0.97	36	66.66
Other, mixed, or unsp. Drug abuse, unsp. Use	Prison	2356	2043	204.53	825	148	358	2.25	51	62.57
Other, mixed, or unsp. Drug abuse, unsp. Use	Intoxications	2356	2663	266.61	889	148	462	2.91	50	60.56
Suicidal ideation	Section XII	1820	3045	235.49	1299	127	403	2.17	81	54.69
Other, mixed, or unsp. Drug abuse, unsp. Use	Undomiciled	2356	2357	235.97	964	148	408	2.57	55	54.50
Other, mixed, or unsp. Drug abuse, unsp. Use	Opioid dependence	2356	1625	162.69	841	148	195	1.23	44	53.86
Suicidal ideation	Schizoaffective schizophrenia	1820	676	52.28	223	127	118	0.64	21	52.75
Other, mixed, or unsp. Drug abuse, unsp. Use	Sober	2356	3667	367.12	1329	148	723	4.55	76	52.29
Other, mixed, or unsp. Drug abuse, unsp. Use	Unspecified bipolar disorder	2356	3488	349.20	932	148	699	4.40	49	48.53
Other, mixed, or unsp. Drug abuse, unsp. Use	Schizoaffective schizophrenia	2356	676	67.68	172	148	118	0.74	15	46.44
Opioid abuse, unspec. Use	Sober	1305	3667	203.35	710	78	723	2.40	42	46.09
Other, mixed, or unsp. Drug abuse, unsp. Use	Methadone	2356	2992	299.54	1165	148	653	4.11	69	45.55
Borderline personality	Methadone	582	2992	74.00	139	35	653	0.97	14	43.59
Opioid abuse, unspec. Use	Living on the street	1305	1113	61.72	293	78	154	0.51	18	43.28
Opioid type dependence, continuous use	Drug seeking	710	463	13.97	96	50	51	0.11	9	37.61
Suicidal ideation	Suicidality	1820	2546	196.90	1057	127	380	2.05	58	35.84
Other, mixed, or unsp. Drug abuse, unsp. Use	Cluster b	2356	495	49.56	175	148	43	0.27	10	35.70
Unspec. Neurotic disorder	Opioid dependence	1003	1625	69.26	191	72	195	0.60	12	35.48

A high IH value indicates that the relationship between *A* and *B* changes significantly between case and non-case populations.

**Table 5:** Structured-unstructured feature pairs *AB* with high interaction heterogeneity (IH), where *A* is a strong *protective factor* against suicide.

Features		Cases				Non-cases				
Structured (*A*)	Unstructured (*B*)	*A*	*B*	*AB* expected	*AB* actual	*A*	*B*	*AB* expected	*AB* actual	IH
Screening mammogram for malignant neoplasm of breast	Imp. Cont. Dis.	89	661	2.50	51	2091	3658	325.03	875	110.08
Annual Exam	Imp. Cont. Dis.	171	661	4.80	81	2596	3658	403.53	1249	94.20
Screening mammogram for malignant neoplasm of breast	vacuuming	89	231	0.87	25	2091	1546	137.37	374	93.77
Screening digital breast tomosynthesis, bilateral	Imp. Cont. Dis	103	661	2.89	46	1656	3658	257.41	730	71.63
Encounter for screening, unspec.	Imp. Cont. Dis	55	661	1.54	30	809	3658	125.75	344	66.36
Screening digital breast tomosynthesis, bilateral	vacuuming	103	231	1.01	23	1656	1546	108.79	332	62.36
Encounter for screening for malignant neoplasm of colon	Imp. Cont. Dis	61	661	1.71	31	1399	3658	217.46	620	57.69
Screening mammogram for malignant neoplasm of breast	Imp. Cont. Dis	89	2019	7.64	80	2091	10987	976.24	1765	53.97
Pure hypercholesterolemia, unsp.	Imp. Cont. Dis	64	661	1.80	30	1328	3658	206.43	596	49.89
Screening digital breast tomosynthesis, bilateral	Imp. Cont. Dis	103	2019	8.84	82	1656	10987	773.15	1422	44.84
Annual Exam	vacuuming	171	231	1.68	23	2596	1546	170.54	423	44.53
Physical therapy evaluation low complex 20 mins	Imp. Cont. Dis	36	661	1.01	22	678	3658	105.39	325	44.29
Screening, malig. neopl. colon	vacuuming	61	231	0.60	14	1399	1546	91.91	269	43.32
Screening, malig. neopl. breast	Imp. Cont. Dis	30	661	0.84	18	571	3658	88.76	272	36.53
Other hemorrhoids	Imp. Cont. Dis	37	661	1.04	17	559	3658	86.89	236	33.29
Age-related osteoporosis without current pathological fracture	Imp. Cont. Dis	32	661	0.90	18	549	3658	85.34	271	32.33
Asymptomatic menopausal state	vacuuming	20	231	0.20	7	387	1546	25.42	81	29.70
Other melanin hyperpigmentation	vacuuming	25	231	0.25	8	699	1546	45.92	156	29.59
Screening, unspecified	Imp. Cont. Dis	55	2019	4.72	46	809	10987	377.70	692	29.58
Mod sed same phys/qhp each addl 15 mins	Imp. Cont. Dis	28	661	0.79	13	822	3658	127.77	329	28.45

A high IH value indicates that the relationship between *A* and *B* changes significantly between case and non-case populations. Among the unstructured concepts, “Imp. Cont. Dis” refers to impulse-control disorder, and “vacuuming” refers to use of hallucinogenic and psychoactive drugs derived from psilocybin mushrooms.

Table 5 shows feature pairs in which the structured feature *A* was associated with lower suicide risk (i.e., *A* occurred less frequently in the case cohort than in the non-case cohort). These include concepts such as annual exams, mammograms, and tumor screenings that are associated with NLP concepts such as impulse-control disorder and use of hallucinogenic and psychoactive drugs derived from psilocybin mushrooms (referred to as “vacuuming” in informal parlance). In many cases, structured codes such as mammograms and tumor screenings are confounded with older age which is protective of suicide attempt risk. Hence lower suicide attempt risk associated with interaction of these structured variables with high-risk concepts such as impulse-control disorder and hallucinogenic drug use is to be expected. (In Tables 4 and 5, “*AB* Expected” corresponds to *E*[*a*_*i*_] used in computation of the *T*_*i*_ statistic defined above.)

As described above, interaction heterogeneity (IH) provides a summary measure of the difference in the overall shape of the contingency tables between case and non-case populations. In order to provide a more intuitive understanding of IH, Tables 6 and 7 provide illustrative examples of contingency tables for two structured-unstructured feature pairs *AB*: One with a high IH value of 77.55 (“Other, mixed, or unspecified drug abuse, unspecified use” & “suicide attempts”), and the other with a low IH value of 3.95 (“Opioid abuse, unspecified use” & “junk (heroin)”). For simplicity, we refer to the number of individuals who had both *A* and *B* in the cases cohort as *AB*_cases_, and to the number of people who had *A* but did not have *B* in the cases cohort as *A*~*B*_cases_, and so forth.

**Table 6:** Contingency tables for the structured-unstructured pair “Other, mixed, or unspecified drug abuse, unspecified use” (*A*) and “suicide attempts” (*B*).

Cases			Non-cases		
Concept	*B*: 1	*B*: 0	Concept	*B*: 1	*B*: 0
*A*: 1	0.0401	0.0541	*A*: 1	0.0021	0.004
*A*: 0	0.1095	0.7376	*A*: 0	0.0204	0.9150

This feature pair has a high interaction heterogeneity (IH) value of 77.55. Values shown are proportions of the total number of samples (23,566) for each bin.

**Table 7:** Contingency tables for the structured-unstructured pair “Opioid abuse, unspecified use” (*A*) and “junk (heroin)” (*B*).

Cases			Non-cases		
Concept	*B*: 1	*B*: 0	Concept	*B*: 1	*B*: 0
*A*: 1	0.0443	0.0079	*A*: 1	0.0022	0.0010
*A*: 0	0.1071	0.7820	*A*: 0	0.0297	0.9085

This feature pair has a low IH value of 3.95. Values shown are proportions of the total number of samples (23,566) for each bin. The differences between the two distributions are smaller in Table 7 than in Table 6, resulting in a lower IH value.

The values for *AB*_cases_ and *AB*_non-cases_ are similar for both pairs of contingency tables (Tables 6 and 7), as are the values for ~*AB*_cases_ and ~*AB*_non-cases_. However, the differences between *A*~*B*_cases_ and *A*~*B*_non-cases_, and the differences between ~*A*~*B*_cases_ and ~*A*~*B*_non-cases_ are greater in Table 6 than in Table 7. Thus, the overall shape of the contingency table in Table 6 changes more between case and non-case populations than the contingency in Table 7. This yields a larger IH value for Table 6 and a smaller IH value for Table 7, indicating that the interaction of concepts in Table 6 is more strongly associated with the suicide-attempt outcome.

**Fig. 5:**
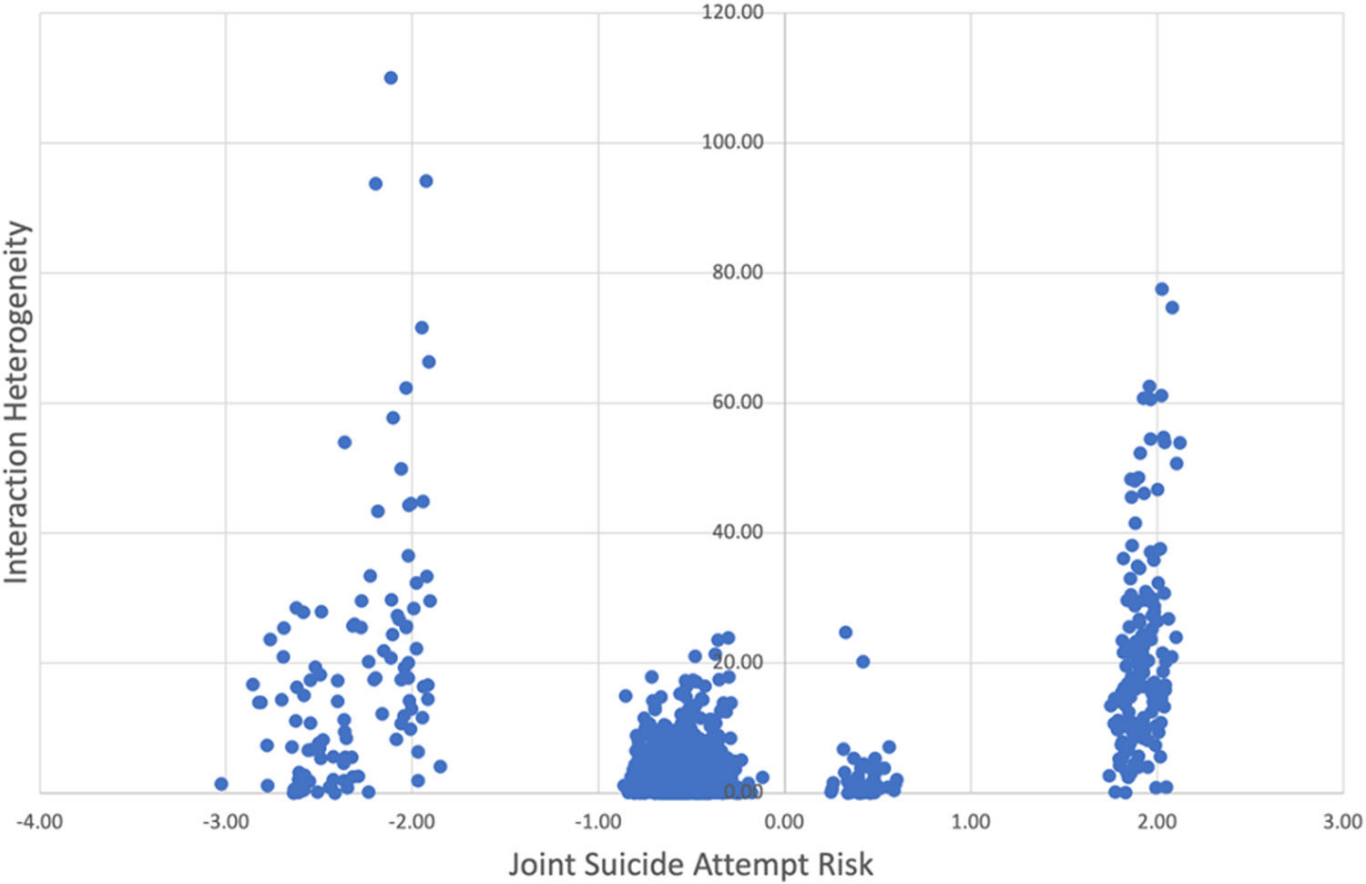
Interaction heterogeneity versus joint suicide risk. A comparison of joint suicide attempt risk and interaction heterogeneity. Each data point corresponds to a structured-unstructured feature pair *AB*. The *x*-axis shows the joint suicide risk of features *A* and *B*, defined as the log of the ratio of the expected joint occurrences of *AB* in the case vs. non case cohorts. The *y*-axis shows the interaction heterogeneity, a measure of how much the interaction between *A* and *B* differs between case and non-case cohorts. The plot shows that feature pairs with similar joint suicide attempt risk can have very different interaction heterogeneity.

In order to study the difference between IH and more traditional measures of risk, Fig. 5 plots IH versus the *joint suicide attempt risk* of features *A* and *B* (defined as the log of the ratio of the expected joint occurrences of *AB* in the case vs. non-case cohorts). As mentioned, IH is a measure of whether the *interaction* between features *A* and *B* differs significantly between case and non-case cohorts. The *joint suicide attempt risk* provides a summary measure of association between the features and the outcome, reflecting the difference in the number of occurrences of *A* and *B* between case and non-case cohorts. (To reduce noise, we only included feature pairs *AB* with at least 10 joint occurrences in either case or non-case cohorts.) Figure 5 shows that many feature pairs with similar joint suicide risk have a large variation in IH—highlighting the fact that IH can reveal variation in feature interactions that the ratio of expected occurrences does not capture.

This is illustrated further in Tables 8 and 9, which presents interactions that correspond to the rightmost cluster in Fig. 5 (i.e., feature pairs with joint suicide risk between 1.7 and 2.3). Within this cluster, Table 8 presents the 20 feature interactions with the highest values of IH, and Table 9 presents the 20 feature interactions with the lowest values of IH. Although the joint suicide risk values are approximately the same in both tables, we see that the nature of interactions is different between Tables 8 and 9. Table 8 contains mostly general substance-abuse structured features (e.g., “Other, mixed, or unspecified drug abuse, unspecified use”), while Table 9 includes specific substance-abuse structured features such as cocaine, methadone, barbiturate, and opioid consumption. Furthermore, the substance abuse codes in Table 8 interact mostly with non-substance-abuse unstructured features such as “lack of domicile”, “schizophrenia” and “imprisonment”, while the substance-abuse codes in Table 9 interact mostly with other substance-abuse-related unstructured features—most prominently, heroine and thioridazine. Thus, interactions between features that are near-synonyms show less difference between case and non-case cohorts than interactions between features that are more heterogeneous.

**Table 8:** Structured-unstructured feature pairs *A*-*B* with high interaction heterogeneity (IH) values.

Structured feature (*A)*	Unstructured feature (*B*)	Joint suicide attempt risk	IH
Other, mixed, or unspecified drug abuse, unspecified use	Suicide attempts	2.02	77.55
Other, mixed, or unspecified drug abuse, unspecified use	Section XII	2.08	74.72
Other, mixed, or unspecified drug abuse, unspecified use	Living on the street	2.06	66.66
Other, mixed, or unspecified drug abuse, unspecified use	Prison	1.96	62.57
Other, mixed, or unspecified drug abuse, unspecified use	Undomiciled	2.02	61.18
Other, mixed, or unspecified drug abuse, unspecified use	Intoxications	1.96	60.56
Suicidal ideation	Section XII	2.03	54.69
Other, mixed, or unspecified drug abuse, unspecified use	Undomiciled	1.96	54.50
Other, mixed, or unspecified drug abuse, unspecified use	Opioid dependence	2.12	53.86
Suicidal ideation	Schizoaffective schizophrenia	1.91	52.75
Other, mixed, or unspecified drug abuse, unspecified use	Sober	1.91	52.29
Opioid abuse, unspecified use	Methadone	2.02	48.85
Other, mixed, or unspecified drug abuse, unspecified use	Unspecified bipolar disorder	1.90	48.53
Suicidal ideation	Delusions	1.86	48.32
Other, mixed, or unspecified drug abuse, unspecified use	Methadone	2.00	46.72
Other, mixed, or unspecified drug abuse, unspecified use	Schizoaffective schizophrenia	1.96	46.44
Opioid abuse, unspecified use	Sober	1.93	46.09
Other, mixed, or unspecified drug abuse, unspecified use	Methadone	1.86	45.55
Cocaine abuse, unspecified use	Methadone	1.97	43.78
Borderline personality	Methadone	1.88	43.59

The joint suicide attempt risk of features *A* and *B* is defined as the log of the ratio of the expected joint occurrences of *AB* in the case vs. non case cohorts.

**Table 9:** Structured-unstructured feature pairs *A*-*B* with low interaction heterogeneity (IH) values.

Structured feature (*A*)	Unstructured feature (*B*)	Joint suicide risk	IH
Opioid type dependence, continuous use	Hearing voices	2.03	0.05
Opioid type dependence, continuous use	Suicidality	1.98	0.05
Methadone tab 40 mg	Junk (heroin)	1.73	0.05
Barbiturate and similarly acting sedative or hypnotic abuse, unspecified use	Mugged (assault)	1.96	0.04
Unspecified neurotic disorder	VH (visual hallucinations)	1.89	0.04
Other, mixed, or unspecified drug abuse, unspecified use	Judgment impaired	2.12	0.03
Barbiturate and similarly acting sedative or hypnotic abuse, unspecified use	Prison	2.04	0.03
Opioid type dependence, continuous use	Junk (heroin)	1.83	0.02
Cocaine abuse, unspecified use	Blackouts	1.88	0.02
Methadone tab 40 mg	Junk (heroin)	1.83	0.02
Opioid type dependence, continuous use	Thioridazine	1.99	0.02
Barbiturate and similarly acting sedative or hypnotic abuse, unspecified use	Junk (heroin)	2.11	0.01
Acute alcoholic intoxication in alcoholism, continuous drinking behavior	Hallucinosis	1.99	0.01
Suicidal ideation	Crack	2.02	0.01
Methadone tab 40 mg	Stolen	1.73	0.01
Unspecified neurotic disorder	Sexual assaults	1.81	0.01
Depressive Neuroses (MS v24)	Sober	1.96	0.00
Depressive Neuroses (MS v24)	Prison	2.01	0.00
Unspecified neurotic disorder	VH	1.85	0.00
Cocaine abuse, continuous use	VH	1.95	0.00

The joint suicide attempt risk of features *A* and *B* is defined as the log of the ratio of the expected joint occurrences of *AB* in the case vs. non case cohorts.

## Discussion

We found that models trained only on features derived from structured-data perform better than models trained only on features derived from unstructured data. The performance gap between models trained with structured data and those trained with unstructured data is quite small, considering the compact size of the unstructured data.

Combining unstructured data with structured data provided almost no performance benefit with the NBC model, whereas the BRFC model showed a significant increase in AUC. The fact that the NBC model only negligibly benefitted from the addition of NLP concepts is not surprising; while interactions between structured and unstructured features could contain useful signals, NBCs assume conditional independence among features, and so cannot exploit these interactions to improve predictive performance. On the other hand, BRFCs are designed to capture interactions between features, and are thus able to deliver a significant improvement in predictive performance. Indeed, examining trees in the BRFC model, we found many examples where splits based on NLP concepts were either preceded or followed by structured-data-based splits, bearing evidence that the BRFC models captured useful structured-unstructured interactions.

Structured-unstructured feature pairs whose interactions differed most between suicidal and non-suicidal populations were those that described heterogeneous pairs of general concepts, rather than pairs of similar concepts. In particular, interactions between concepts related to mental health issues, drug abuse, excessive alcohol consumption, and psychiatric disorders were predictive of suicide risk. Although links between these concepts have been previously established, features derived from unstructured data further strengthen these associations. Unstructured data also helps capture complementary information about the well-being of patients that structured data may not provide: The interaction of structured concepts with concepts derived from unstructured data for environmental risk factors (e.g., “living on the street”, “undomiciled”, “prison”), services that occur outside the health system (e.g., “methadone maintenance”), and diagnoses such as “delusions” and “impulse-control disorder” were found to be highly predictive of suicide. Similarly, interactions between medical screenings and general examinations were observed to be protective of suicide risk, although it is unclear whether it was the examinations themselves or other confounding factors related to the examinations that were protective of suicide risk. As seen from the examples in Table 1, many patients who had NLP codes recorded for some concepts did not have the corresponding structured codes in their EHR records which shows that unstructured data can help capture information about a patient that structured data misses. Such insights into the changing nature of feature interactions between case and non-case cohorts can help to improve predictive performance and provide a deeper understanding of clinical risk.

This study is subject to a number of limitations. We analyzed 20 years of longitudinal healthcare data from a single healthcare system including hospital admissions, observational stays, emergency department visits, and outpatient encounters. Visits outside this geographical setting, time period, and network of hospitals were not included, and therefore this study dataset may be missing some encounters which could have potentially been useful for predicting suicide attempts. Moreover, some of these excluded visits may have been for suicidal behavior, meaning that some patients may have been incorrectly identified as non-case subjects or correctly identified as case subjects but given incorrect onset times. For patient diagnoses, we included both ICD-9 and ICD-10 codes since both encoding standards were used in the RPDR during the last 20 years. Due to this, there are some concepts for which both ICD-9 and ICD-10 definitions have been included in the dataset, adding extra computational burden. Since the goal of this research was to investigate properties of structured and unstructured data, we compared predictive performance of NBCs and BRFCs, which are relatively easy to interpret. To achieve a potentially superior predictive model, it would also be worthwhile to consider other modeling approaches such as XGBoost, neural networks, and support vector machines, as well as complex feature selection techniques such as PCA and t-SNE. However, these modeling methods are more difficult to interpret, making them less suitable for the present study. They are potential avenues for future work.

Another limitation is that suicide attempt risk predictions were performed only on the penultimate visits prior to a suicide attempt. This was done to reduce the complexity and computational burden of the prediction task while allowing us to focus on differences between structured and unstructured features. As a result, the specific models developed here are designed to predicting risk in later visits of patients and may not predict suicide risk sufficiently in advance if used in earlier visits. Predictive models trained for practical purposes would be designed for predicting at any point during the patient’s longitudinal history. One approach for doing this with random forests is to sample random visits in the patient’s medical timeline and include cumulative feature history up until that visit as “snapshots.” We have explored such multi-temporal suicide risk predictions with random forests in a separate study^10^.

It is likely that some patients who attempted suicide did not have their suicide attempts recorded in the electronic medical record, either due to failures in diagnosis, errors in recording the suicide attempt, or simply because the patient did not have an encounter with the health system around the suicide attempt. Therefore, the actual misclassification rate of case subjects by our models could be higher than reported. This limitation with case labeling is inherent to any study on suicide risk prediction based on EHR data. Although in this study only the structured codes were used in case definitions, future work can also consider using suicide attempt concepts in clinician notes when labeling cases. This way, NLP concepts would not only expand the feature set of the patients, but also expand the list of cases for training and testing. Given the imbalanced 1/99 case—non-case ratio, this could improve the predictive accuracy of trained models. Future work could also compare the performance of these models on different demographic, socio-economic, and time cohort subgroups. It would also be worthwhile to consider stratifying models by variables known to be confounded with suicide attempt risk such as age.

When developing the models for this study, we constrained ourselves to methods that are relatively easy to interpret. We also tried to keep the complexity of our models and NLP pipeline relatively low for better clinical generalizability. Therefore, our rule-based NLP pipeline and NBC models are highly interpretable. Although the training process of RF models can be obscure due to the randomness and ensembling involved, trees can be easily visualized to justify decisions made by the model during inference. However, if the interpretability constraint were waived, it would be worthwhile to explore other modeling approaches, including deep learning artificial neural network (ANN) models, for both NLP feature extraction and predictive modeling. Although such models are typically harder to interpret, deep learning models have exhibited superior predictive performance compared to statistical and machine learning models in a number of computational tasks. In particular, recent advancements made in recurrent neural networks (RNNs) and attention-based transformer networks have proven great potential in many types of natural language tasks. ANNs are able to extract abstract representations from different modes of input data, without any feature engineering involved and fuse them efficiently for optimal predictive performance. ANNs have already been employed for many medical prediction tasks involving structured and unstructured data. In the suicide prediction workflow described in this study, both our NLP feature extraction pipeline and RF-based risk modeling could be replaced with deep-learning approaches for greater predictive performance. Future work could explore the application of deep learning to NLP feature extraction and prediction of suicide attempt risk^11–16^.

Previous studies have examined the use of unstructured EHR data in clinical prediction models in general, and in suicide prediction models in particular. Tsui et al.^1^ showed that the use of NLP features extracted from clinician notes significantly improved the AUC of an ensemble of extreme gradient boosting models and of a Lasso model over a structured-data only baseline model. Poulin et al. used keywords extracted from unstructured clinician notes to predict suicide risk among US veterans with an accuracy of 65%^4^. Carson et al constructed a random forest model trained on structured and unstructured EHR data of psychiatrically hospitalized adolescents to predict suicidal behavior with an AUC of 0.68^17^.

In the present study, we examined the integration of features derived from unstructured clinician notes into structured-data-based suicide risk prediction models. We showed that a model that assumes independence among variables (NBC) does not significantly benefit from the addition of unstructured features, whereas models such as Balanced Random Forest Classifiers that explicitly capture interactions exhibit performance increases when unstructured features are added. We also proposed and implemented a framework for identifying specific structured-unstructured feature pairs whose interaction patterns differ with respect to a patient’s suicide risk, and thus have the potential to improve predictive performance and increase understanding of clinical risk. Many of the interactions identified are expected, which serves to validate our proposed approach for identifying meaningful interactions that can help further elucidate the risk factors of clinical conditions. These findings and this framework can be used to improve current and future EHR-based clinical prediction models, which are becoming increasingly widespread in clinical settings.

## Methods

### Data processing

We analyzed data from the Mass General Brigham Research Patient Data Registry (RPDR)^18^, an EHR data warehouse covering 4.6 million patients from two large academic medical centers in Boston, MA, USA (Massachusetts General Hospital and Brigham and Women’s Hospital), as well as their affiliated community and specialty hospitals in the Boston area. The RPDR was queried for all inpatient and outpatient visits occurring from 1998 through 2018 by individuals who met the inclusion criteria of: Three or more total visits recorded in the EHR, 30 days or more between the first and last visits, and the existence of at least one encounter after age 10 and before age 90. For each patient, we analyzed all demographic, diagnostic, procedure, laboratory, and medication data recorded at each visit, as well the unstructured clinician notes.

The structured data extracted from the RPDR was retrieved in a format wherein each row corresponded to a specific feature recorded during a specific encounter. All datasets had the following columns: de-identified subject number, encounter number, concept code, concept start date, and site of encounter. Lab results data had a “valueflag” column representing the recorded outcome of a given test (low, normal, high, abnormal, undetermined) which was merged into the lab features column. This transformed each lab feature into multiple “dummy” features. For example, instead of a feature “test_1”, we had five features: “test_1|L”, “test_1|N”, “test_1|H”, “test_1|U”, “test_1|A”. Each NLP concept was appended with the mention type of the concept (positive; negative; family history; negative family history), which quadrupled the number of NLP features. The “Concept start date” column was used for filtering patients by age along with demograpihcs data, and for removing data recorded following the first suicide attempt for cases.

### Natural language processing

In order to derive features from the unstructured clinician notes, we created a custom lexicon of suicide-relevant and psychiatric concepts using a variety of approaches including: (1) selecting signs and symptoms, and mental and behavioral process semantic types from the Unified Medical Language System (UMLS)^19^; (2) mapping DSM symptoms and concepts from structured instruments^20^; (3) automatically extracting features from public sources including Wikipedia and MedScape; (4) incorporating RDoC domain matrix terms^20^; (5) selecting predictive features from coded suicide attempt prediction models^21^; and (6) manual annotation of terms by expert clinicians. This lexicon was linked to UMLS concepts and included 480 distinct semantic concepts and 1,273 tokens or phrases. Using this lexicon, we ran the HiTex^22^ NLP named-entity extraction pipeline to identify concepts in over 120 million clinical notes. For each note, we identified the presence of a concept (e.g., symptom, disease, mental process) and further tagged concepts as negated (NEG), family history mention (FH) or negated family history (NFH). For negation and family history pipeline components, we utilized the ConText algorithm^23^.

### Case definition

We have previously described the development of an EHR-based case definition for suicide^9^. In summary, with the help of three expert clinicians, we identified codes from *International Classification of Diseases, Ninth Revision* (ICD-9) and *International Classification of Diseases, Tenth Revision* (ICD-10) that reliably captured suicide attempts with a positive predictive value (PPV) of greater than 0.70. Subjects having at least one of these codes were included in the case population. For cases, we also removed all data following the first suicide attempt (the index event) and made predictions at the penultimate visit prior to the index event. For the purpose of this study, the case definition was based solely on structured diagnostic information and did not include information derived from the clinician notes when classifying individuals as cases versus non-cases.

### Model training

We split our data into training and testing sets with a 70/30 ratio, respectively. For each individual, we included all visits available in that patient’s EHR. For individuals labeled as cases, we included only visits up to and including the penultimate visit prior to the visit on which the individual first met the case definition. This restriction was not applied to non-cases. We applied two modeling approaches for suicide prediction. The first was a Naive Bayes Classifier (NBC) model, described in detail elsewhere^24^. NBCs are a subclass of Bayesian networks that assume strong conditional independence of all input features, greatly reducing model complexity^25^. NBCs have been shown to be well-suited for clinical decision support tasks and are highly scalable and interpretable; they compute a risk score for each concept using the odds ratios of its prevalence in case and non-case populations, ignoring interactions with other variables. During validation, the NBC risk scores for each concept in a patient’s visit history were added together to compute a cumulative suicide risk measure for the subject. If a patient had multiple instances of the same predictor over multiple visits, that predictor was counted multiple times at different visits of the patient. The NBC model was trained using *R* version 3.6.0 and the *R* packages *pROC* and *tidyverse*.

The second modeling approach was a Balanced Random Forest Classifier (BRFC)^26^, which unlike NBCs is capable of capturing interactions between features. Balanced Random Forests are an extension of Random Forest^27^ models, which work well with label-imbalanced datasets. Due to computational constraints, the BRFCs were trained and tested on a smaller subset of 140,000 subjects of the RPDR data. The occurrence rate of suicide attempts in our dataset is very low, at about 1%, resulting in low positive predictive values (PPV) on test sets with regular Random Forests. BRFCs balance the classes by either downsampling the majority class, upsampling the minority class, or resampling both classes with replacement during bootstrap draws until a specified ratio of classes is met. During the sampling of training data, we ensured that the proportion of cases was lifted from 1% to around 12%. The test set was left intact with the natural 1% suicide attempt rate. The data pipeline for arriving at training and testing sets for all described models is illustrated in Fig. 2.

For selecting the parameters of the model, we performed a grid search with 5-fold cross-validation on the BRFC parameter space. Based on the grid search results, we arrived at a model with 30 trees, 50% of all features sampled for each tree, bootstrap sample size equal to the total number of samples, and 1:4 ratio of case to non-cases in every bootstrap sample, achieved with random undersampling of the majority class. Even after undersampling non-cases to 1:4 case:non-case ratio, the size of bootstrap samples remained sufficiently large due to the relatively high case prevalence (12%) in the training data. We used *Python* version 3.6.9 with the libraries *scikit-learn, imblearn, numpy*, *pandas*, and *matplotlib*. The packages *imblearn and scikit-learn* were useful for training and testing balanced random forests. Libraries *numpy* and *pandas* were helpful for data transformations and analyses. Paper visualizations were produced using *matplotlib*.

We used area under the receiver operating characteristic curve (AUC) as the primary predictive performance metric. In order to create confidence intervals and enable comparison of AUC values of different models, we used the percentile bootstrapping method with a simulation size of 1,000. We also measured PPV and sensitivity over a range of specificities. Since the primary goal of our work was to investigate properties of the NLP dataset rather than to build an optimal predictive model, we maximized simplicity in the study design: All predictions were made at the visit prior to the first suicide attempt for cases, and the last visit recorded for non-cases.

### Contingency analysis

In order to better understand the interactions between structured and unstructured data, we performed a separate contingency analysis to identify interactions between structured and unstructured features that differed substantially between case and non-case populations. To account for possible effects of sample size differences between case and non-case populations, we randomly sampled two equal cohorts—one with 23,566 cases and the other with 23,566 non-cases. (These cohorts were sampled from the original dataset before training and testing splits were made.) To simplify analysis, we counted each feature only at its first occurrence for each subject.

For simplicity in the following discussion, we will refer to a feature derived from structured data as *A*, and a feature derived from NLP of unstructured data as *B*. For each feature pair *A-B*, we computed contingency tables for both case and non-case populations (Table 10). To measure the strength of association between feature *A* and feature *B* within each cohort, we performed a Chi-squared test of independence. The null hypothesis was that *A* is independent of *B*, while the alternative hypothesis was that there is an association between *A* and *B*. Equation (1) shows the computation of the statistic *T*_*i*_ for both case and non-case populations:

1 $$\begin{array}{l}T_i = {\sum} {\frac{{a_i - {{E}}\left[ {a_i} \right]}}{{{{E}}\left[ {a_i} \right]}}} ,{{E}}\left[ {a_i} \right] = \frac{{\left( {a_i + c_i} \right)\left( {a_i + b_i} \right)}}{n},n = a_i + b_i + c_i + d_i\\ T_i \sim \chi _1^2\end{array}$$

where *a, b, c*, and *d* are as defined in Table 10. Under the null hypothesis, *T*_*i*_ follows a Chi-squared distribution with one degree of freedom. This value can be used to compute *p*-values from the Chi-squared quantile function.

**Table 10:** Contingency tables of structured-unstructured concept pairs *A-B*, for case and non-case cohorts.

Cases			Non-cases		
Concept	*B*: 1	*B*: 0	Concept	*B*: 1	*B*: 0
*A*: 1	*a*_1_	*b*_1_	A: 1	*a*_0_	*b*_0_
*A*: 0	*c*_1_	*d*_1_	A: 0	*c*_0_	*d*_0_

In order to determine whether the interactions between feature *A* and feature *B* differed between case and non-case populations, we used Woolf’s method for testing for homogeneity^28^. The null hypothesis was that the odds ratios computed on each of the case and non-case populations were equal, while the alternative hypothesis was that these differed significantly. We calculated Woolf’s test statistic (*X*^2^_HOM_) as shown in Eq. 2:

2 $$\begin{array}{c}\log \left( {\widehat {OR}_i} \right) = \log \left( {\frac{{a_id_i}}{{b_ic_i}}} \right)\\ \left[ {{{{\mathrm{Var}}}}\left( {\log \left( {\widehat {OR}_i} \right)} \right)} \right]^{ - 1} = w_i = \left( {\frac{1}{{a_i}} + \frac{1}{{b_i}} + \frac{1}{{c_i}} + \frac{1}{{d_i}}} \right)^{ - 1}\\ \overline {\log OR} = \frac{{\mathop {\sum}\nolimits_{i = 1}^k {w_i} \log \left( {\widehat {OR}_i} \right)}}{{\mathop {\sum}\nolimits_{i = 1}^k {w_i} }}\\ X_{{\rm{HOM}}}^2 = \mathop {\sum}\limits_{i = 1}^k {w_i} \left( {\log \widehat {OR}_i - \overline {\log OR} } \right)^2\\ X_{{\rm{HOM}}}^2\mathop { \sim }\limits^{{{{\mathrm{asym}}}}} \chi _{k - 1}^2\end{array}$$

For *k* = *0*, under the null hypothesis, *X*^2^_HOM_ follows a Chi-squared distribution with one degree of freedom. For clarity, we will refer to Woolf’s test statistic *X*^2^_HOM_ as *Interaction Heterogeneity* (IH). Interaction heterogeneity provides a summary measure of the difference in the overall shape of the contingency table between case and non-case populations.

Next, we examined the joint distribution *p*(*AB*|*Y*), conditional on the case variable *Y* (suicide vs. non-suicide). Using Bayes’ rule, this distribution can be used to derive the more clinically interesting distribution *p*(*Y*|*AB*)—specifically *P*(*Y* = 1|*A* = 1*, B* = 1)—which is the probability of the patient attempting suicide in the future given that the patient has both features *A* and *B*:

3 $$\begin{array}{l}P\left( {Y\mid A,B} \right) = \frac{{P\left( {A\mid Y,B} \right)P\left( {Y\mid B} \right)}}{{P\left( {A\mid B} \right)}}\\ P\left( {A = 1\mid Y = 1,B = 1} \right) = \frac{{a_1}}{{a_1 + b_1}}\\ P\left( {Y = 1\mid B = 1} \right) = \frac{{a_1 + b_1}}{{a_0 + b_0 + a_1 + b_1}}\\ P\left( {A = 1\mid B = 1} \right) = \frac{{a_1 + a_0}}{{a_1 + b_1 + a_0 + b_0}}\\ P\left( {Y = 1\mid A = 1,B = 1} \right) = \frac{{\left( {\frac{{a_1}}{{a_1 + b_1}}} \right)\left( {\frac{{a_1 + b_1}}{{a_0 + b_0 + a_1 + b_1}}} \right)}}{{\frac{{a_1 + a_0}}{{a_1 + b_1 + a_0 + b_0}}}}\end{array}$$

The variables *a*_*i*_*, b*_*i*_*, c*_*i*_*, d*_*i*_ shown in Eq. 3 are as in Table 10, except that the entries in the contingency table of cases have been divided by 100 to reflect the 1/99 case-non-case ratio encountered in the clinical population. Thus, using Woolf’s method, we are able to identify specific structured-unstructured feature interactions that are most different between case and non-case cohorts, and thus have the most potential for improving predictive performance.

Combining the above methods, we assembled a list of structured-unstructured feature pairs *AB* in which: (1) Both *A* and *B* were among the top 200 most important features as ranked by the absolute value of the NBC feature risk scores; (2) the joint occurrence of *A* and *B* were significantly different from the expected value under the null within both case and non-case cohorts, as measured using the Chi-squared statistic *T*_*i*_; and (3) the interaction between *A* and *B* was significantly different (heterogeneous) between the case population and the non-case population—as measured by interaction heterogeneity (IH). For ease of interpretation, we included only unstructured features that were either “positive” or “positive family history” mentions, and excluded “negative” and “negative family history” mentions.

Since the goal of this analysis was not to simply find meaningful interactions in the dataset, but rather to identify meaningful interactions between structured and unstructured features, we performed the contingency analysis on structured-unstructured feature pairs, but not on structured–structured or unstructured–unstructured feature pairs.

### Ethics

This research was approved by the Mass General Brigham Institutional Review Board, along with an IRB reliance agreement from the Boston Children’s Hospital Institutional Review Board.

### Reporting summary

Further information on research design is available in the Nature Research Reporting Summary linked to this article.

## Supplementary information

Reporting Summary

## Data Availability

The data used in this study cannot be made available due to restrictions relating to the use of EHR data. This restriction also prevents the data from being made available upon request from the authors.

## Code availability

The code used in this study is available from the authors upon request.
