# Supplementary material for: Predictive structured–unstructured interactions in EHR models: A case study of suicide prediction
Source: NPJ Digit Med. 2022 Jan 27;5:15. doi: 10.1038/s41746-022-00558-0 (PMC8795240; doi:10.1038/s41746-022-00558-0)
Supplement: Supplementary file 1 — Reporting Summary [file 41746_2022_558_MOESM1_ESM.pdf]

nature portfolio

Corresponding author(s): Ben Y. Reis

Last updated by author(s): Oct 21, 2021

Reporting Summary

Nature Portfolio wishes to improve the reproducibility of the work that we publish. This form provides structure for consistency and transparency in reporting. For further information on Nature Portfolio policies, see our Editorial Policies and the Editorial Policy Checklist.

Statistics

For all statistical analyses, confirm that the following items are present in the figure legend, table legend, main text, or Methods section.

n/a	Confirmed	
☐	☒	The exact sample size (*n*) for each experimental group/condition, given as a discrete number and unit of measurement
☐	☒	A statement on whether measurements were taken from distinct samples or whether the same sample was measured repeatedly
☐	☒	The statistical test(s) used AND whether they are one- or two-sided<br>*Only common tests should be described solely by name; describe more complex techniques in the Methods section.*
☐	☒	A description of all covariates tested
☒	☐	A description of any assumptions or corrections, such as tests of normality and adjustment for multiple comparisons
☐	☒	A full description of the statistical parameters including central tendency (e.g. means) or other basic estimates (e.g. regression coefficient) AND variation (e.g. standard deviation) or associated estimates of uncertainty (e.g. confidence intervals)
☒	☐	For null hypothesis testing, the test statistic (e.g. *F*, *t*, *r*) with confidence intervals, effect sizes, degrees of freedom and *P* value noted<br>*Give P values as exact values whenever suitable.*
☒	☐	For Bayesian analysis, information on the choice of priors and Markov chain Monte Carlo settings
☒	☐	For hierarchical and complex designs, identification of the appropriate level for tests and full reporting of outcomes
☒	☐	Estimates of effect sizes (e.g. Cohen's *d*, Pearson's *r*), indicating how they were calculated

Our web collection on statistics for biologists contains articles on many of the points above.

Software and code

Policy information about availability of computer code

Data collection	We used Python version 3.6.9 with the libraries scikit-learn, imblearn, numpy, pandas, and matplotlib. The packages imblearn and scikit-learn were useful for training and testing balanced random forests. Libraries numpy and pandas were helpful for data transformations and analyses. Paper visualizations were produced using matplotlib.
Data analysis	We used Python version 3.6.9 with the libraries scikit-learn, imblearn, numpy, pandas, and matplotlib. The packages imblearn and scikit-learn were useful for training and testing balanced random forests. Libraries numpy and pandas were helpful for data transformations and analyses. Paper visualizations were produced using matplotlib.

For manuscripts utilizing custom algorithms or software that are central to the research but not yet described in published literature, software must be made available to editors and reviewers. We strongly encourage code deposition in a community repository (e.g. GitHub). See the Nature Portfolio guidelines for submitting code & software for further information.

Data

Policy information about availability of data

All manuscripts must include a data availability statement. This statement should provide the following information, where applicable:

- Accession codes, unique identifiers, or web links for publicly available datasets
- A description of any restrictions on data availability
- For clinical datasets or third party data, please ensure that the statement adheres to our policy

The data used in this study cannot be made publicly available due to restrictions relating to the use of electronic health record data.

Field-specific reporting

Please select the one below that is the best fit for your research. If you are not sure, read the appropriate sections before making your selection.

☒ Life sciences ☐ Behavioural & social sciences ☐ Ecological, evolutionary & environmental sciences

For a reference copy of the document with all sections, see nature.com/documents/nr-reporting-summary-flat.pdf

Life sciences study design

All studies must disclose on these points even when the disclosure is negative.

Sample size	The RPDR was queried for all inpatient and outpatient visits occurring from 1998 through 2018 by individuals who met the inclusion criteria of: Three or more total visits recorded in the EHR, 30 days or more between the first and last visits, and the existence of at least one encounter after age 10 and before age 90. For each patient, we analyzed all demographic, diagnostic, procedure, laboratory, and medication data recorded at each visit, as well the unstructured clinician notes.
Data exclusions	All patients not meeting the inclusion criteria above.
Replication	N/A
Randomization	N/A
Blinding	N/A

Reporting for specific materials, systems and methods

We require information from authors about some types of materials, experimental systems and methods used in many studies. Here, indicate whether each material, system or method listed is relevant to your study. If you are not sure if a list item applies to your research, read the appropriate section before selecting a response.

Materials & experimental systems

n/a	Involved in the study	
☒	☐	Antibodies
☒	☐	Eukaryotic cell lines
☒	☐	Palaeontology and archaeology
☒	☐	Animals and other organisms
☐	☒	Human research participants
☐	☒	Clinical data
☒	☐	Dual use research of concern

Methods

n/a	Involved in the study	
☒	☐	ChIP-seq
☒	☐	Flow cytometry
☒	☐	MRI-based neuroimaging

Human research participants

Policy information about studies involving human research participants

Population characteristics	The RPDR was queried for all inpatient and outpatient visits occurring from 1998 through 2018 by individuals who met the inclusion criteria of: Three or more total visits recorded in the EHR, 30 days or more between the first and last visits, and the existence of at least one encounter after age 10 and before age 90. For each patient, we analyzed all demographic, diagnostic, procedure, laboratory, and medication data recorded at each visit, as well the unstructured clinician notes.
Recruitment	The RPDR was queried for all inpatient and outpatient visits occurring from 1998 through 2018 by individuals who met the inclusion criteria of: Three or more total visits recorded in the EHR, 30 days or more between the first and last visits, and the existence of at least one encounter after age 10 and before age 90. For each patient, we analyzed all demographic, diagnostic, procedure, laboratory, and medication data recorded at each visit, as well the unstructured clinician notes.
Ethics oversight	This research was approved by the Mass General Brigham Institutional Review Board, along with an IRB reliance agreement from the Boston Children's Hospital Institutional Review Board.

Note that full information on the approval of the study protocol must also be provided in the manuscript.

Clinical data

Policy information about clinical studies

All manuscripts should comply with the ICMJE guidelines for publication of clinical research and a completed CONSORT checklist must be included with all submissions.

Clinical trial registration	N/A
Study protocol	N/A
Data collection	The RPDR was queried for all inpatient and outpatient visits occurring from 1998 through 2018 by individuals who met the inclusion criteria of: Three or more total visits recorded in the EHR, 30 days or more between the first and last visits, and the existence of at least one encounter after age 10 and before age 90. For each patient, we analyzed all demographic, diagnostic, procedure, laboratory, and medication data recorded at each visit, as well the unstructured clinician notes.
Outcomes	Suicide attempt as defined by the case definition in the Methods section.
